# Profilometry of thin films on rough substrates by Raman spectroscopy

**DOI:** 10.1038/srep37859

**Published:** 2016-12-06

**Authors:** Martin Ledinský, Bertrand Paviet-Salomon, Aliaksei Vetushka, Jonas Geissbühler, Andrea Tomasi, Matthieu Despeisse, Stefaan De Wolf, Christophe Ballif, Antonín Fejfar

**Affiliations:** 1 Laboratory of Nanostructures and Nanomaterials, Institute of Physics, Academy of Sciences of the Czech Republic, v. v. i., Cukrovarnická 10, 162 00 Prague, Czech Republic; 2 PV-Center, Centre Suisse d’Électronique et de Microtechnique, Rue Jaquet-Droz 1, CH-2002 Neuchâtel, Switzerland; 3 École Polytechnique Fédérale de Lausanne (EPFL), Institute of microengineering (IMT), Photovoltaics and Thin Film Electronics Laboratory, Rue de la Maladière 71b, CH-2000 Neuchâtel, Switzerland; 4 King Abdullah University of Science and Technology (KAUST), KAUST Solar Center (KSC), Thuwal, 23955-6900, Saudi Arabia

## Abstract

Thin, light-absorbing films attenuate the Raman signal of underlying substrates. In this article, we exploit this phenomenon to develop a contactless thickness profiling method for thin films deposited on rough substrates. We demonstrate this technique by probing profiles of thin amorphous silicon stripes deposited on rough crystalline silicon surfaces, which is a structure exploited in high-efficiency silicon heterojunction solar cells. Our spatially-resolved Raman measurements enable the thickness mapping of amorphous silicon over the whole active area of test solar cells with very high precision; the thickness detection limit is well below 1 nm and the spatial resolution is down to 500 nm, limited only by the optical resolution. We also discuss the wider applicability of this technique for the characterization of thin layers prepared on Raman/photoluminescence-active substrates, as well as its use for single-layer counting in multilayer 2D materials such as graphene, MoS_2_ and WS_2_.

Accurate thickness control, based on non-destructive measurement methods is essential for the successful integration of a wide range of thin and ultrathin films in an equally wide variety of electronic devices. Spectroscopic ellipsometry (SE)^1^, optical interference contrast or direct observation based on probe methods (e.g. by stylus profilometry or atomic force microscopy) are frequently used to this end, often yielding sub-nm resolution. However, these techniques are usually not applicable for thickness detection of films that were either patterned or deposited on substrates with micron-sized roughness, restrictions that are frequently found in actual devices. Photovoltaic solar cells are no exception to this, with the high-efficiency crystalline silicon (c-Si) heterojunction (SHJ) solar cell as prime example^2^.

SHJ devices use c-Si wafers as optical absorber, usually with textured surfaces in the form of several μm large pyramids to minimize the optical reflection^3^. Untreated, these surfaces are electronically defective, but by deposition of 5–10 nm thin hydrogenated amorphous silicon (a-Si:H) layers they become well passivated^4^,^5^,^6^,^7^. Collection of photo-generated electrons and holes is achieved in these devices by deposition of equally thin n-type and p-type a-Si:H overlayers on opposite wafer surfaces^8^,^9^. To allow external carrier extraction, both surfaces are finally capped by stacks of transparent conductive oxides and metal electrodes. Thanks to the high passivation quality of such a-Si:H-based passivating contacts, SHJ solar cells typically display very high operating voltages^10^. Furthermore, by carefully tuning the thin-film properties – especially their thickness and optical properties – an excellent trade-off between light in-coupling^11^ and carrier extraction^12^ is possible, evidenced by conversion efficiencies as high as 25.1% for large-area devices, reported by Kaneka, Japan^13^. This trade-off can be further relaxed by placing both electron- and hole-collecting contacts at the rear of the device, in an interdigitated back contacted design^14^. The validity of this approach applied to SHJ devices was convincingly proven in 2014 by Panasonic, Japan, by the setting of a new world record conversion efficiency for silicon based solar cells of 25.6%^15^. The particular appeal of this device architecture was recently further underlined by Kaneka, Japan, reporting an update of this record to 26.3%^16^. These outstanding results are a strong case for the argument that the integration of silicon heterojunction contact technology into a back contacted design is the ultimate silicon single-junction device architecture for high efficiencies.

The actual processing of the back-contacted SHJ (BC-SHJ) devices requires accurate patterning of the electron- and hole-collecting regions at the rear of the device into two interdigitated combs, made out of p- and n-type a-Si:H strips, with a typical width of 1–2 mm. Very few details are yet known about the processing complexity needed the make the record devices of Panasonic and Kaneka. In any case, to make this technology relevant for large-scale production environments, simple fabrication methods, not relying on photolithography are a must. To this end, in earlier work, we reported on the patterning of such p- and n-type a-Si:H interdigitated fingers by *in-situ* shadow masking during plasma-enhanced chemical vapor deposition (PECVD)^17^. Using this simple patterning method, we experimentally evidenced that the thickness and overall shape of the a-Si:H combs – which depend on the shadow mask dimensions, among other factors – strongly influence the final BC-SHJ device performance, especially its open-circuit voltage (V_OC_) and fill factor (FF)^18^. However, a straightforward method to characterize the a-Si:H comb morphology has been missing, so far. Indeed, all but one of the above-mentioned standard optical and probe techniques are exclusively restricted to flat surfaces. The notable exception is SE, which was earlier reported to determine the a-Si:H thickness in SHJ solar cells on textured wafers using the so-called tilt measurement configuration^19^. In this case, the random-pyramid textured c-Si wafer is tilted such that one of the pyramid facets is oriented so that it reflects the incident light to the detector. In this way, the standard SE measurement configuration and interpretation may be used. This technique works well for surface structures larger than 10 μm, however for typical 3–8 μm large pyramids the simulation starts to be inaccurate. Moreover, SE cannot provide the needed spatial resolution to map 1 mm wide a-Si:H stripes, as the SE light spot size is usually wider than 1 mm. Cross-sectional scanning electron microscopy may be used instead^17^, but is highly time-consuming, destructive and impractical.

In this article we present a new method for thickness profiling of thin films based on Raman micro-spectroscopy. We demonstrate our technique by probing stripes of thin a-Si:H films deposited on both flat and rough c-Si substrates, the structures found back in industry-relevant devices such as high-efficiency BC-SHJ solar cells. Attenuation of the Raman signal from the silicon wafer by absorption in the overlying amorphous silicon allows mapping its thickness over the whole active area of the solar cell under test. In order to optimize the sensitivity of this technique we used a 442 nm excitation wavelength which is highly absorbed in a-Si:H. In this case the detection limit is below 1 nm and its spatial resolution is only limited by the optical resolution of the used optical set-up (down to 500 nm). The Raman absorption-based measurement method is not restricted to the specific example discussed here, but may be used for *all* thin absorbing films prepared on a Raman/photoluminescence active substrate. Application of this method to detect the thickness of absorbing 2D multi-layer materials^20^ is possible and expected to become of high importance to the nanofabrication community.

## Experimental

Samples were prepared on flat and textured c-Si substrates. The flat substrates were float-zone (FZ) n-type, 280-μm-thick double-side mirror polished c-Si wafers. To prepare the textured substrates, we used (FZ) n-type (100), as-cut c-Si wafers as starting material. These wafers first received a saw-damage removal etching step, followed by alkaline texturing and chemical cleaning. The alkaline texturing forms random pyramids with average dimensions of 4 μm in width and 2 μm in height. The final thickness of the textured wafers was 260 μm.

**Figure 1:**
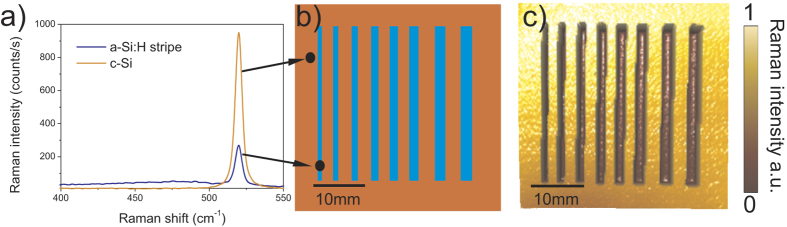
(**a**) Raman spectra measured on the a-Si:H stripe (blue line) and on an uncovered part of the flat c-Si wafer (orange line). (**b**) Sketch of the test sample. (**c**) Corresponding Raman map of c-Si integral intensities, integrated over 505–535 cm^-1^ range.

The shadow mask used during the a-Si:H film deposition was cut in a 280-μm-thick double side-polished c-Si wafer using an IR laser. It consisted of eight 30-mm-long slits with widths from 0.8 mm to 2.2 mm by 0.2 mm steps, This range encompasses the typical widths used in our BC-SHJ devices^17^. A sample prepared using such shadow mask is depicted in Fig. 1b.

Prior to a-Si:H deposition, the samples were shortly dipped into a 5% hydrofluoric acid solution to remove the chemically-grown oxide. They were then loaded into an Octopus II PECVD reactor from INDEOtec SA, the *in-situ* shadow mask being mechanically fixed on top of the samples using fiducials. A 40-nm-thick p-type a-Si:H layer (as measured on flat witness glass samples by SE) was deposited on both samples using a gas mixture of SiH_4_, H_2_, and B(CH_3_)_3_. Note that the deposition time was the same for the flat and the textured substrates.

The Raman spectra were measured in the back-scattering geometry using a micro-spectroscopic Raman setup equipped with aHeCd excitation laser (wavelength of 442 nm, InVia REFLEX, Renishaw) and *xyz* stage allowing mapping of large areas (i.e. hundreds of cm^2^) with high sub μm precision. Raman maps were measured using either a 50× or 5× objective with numerical apertures of 0.5 and 0.05, respectively. The laser spot size was ~ 1 and 10 μm for the 50× and the 5× objective respectively. The intensity of the incident laser was reduced by neutral density filters to several tens of mW in order to prevent local sample heating. The integral Raman intensity of c-Si was calculated in the region 505–535 cm^−1^. A levelling stage, aimed at fine inclination adjustment of the sample, was used to ensure unchanged signal accumulation efficiency over the whole probed area.

## Results

Raman spectra measured on an a-Si:H stripe and on an uncoated part of the c-Si wafer are both plotted in the graph in Fig. 1a. Both spectra reveal a prominent sharp c-Si Raman band at 520 cm^−1^, but their respective intensities differ significantly. The signal acquired on the a-Si:H stripe contains an additional weak broad Raman band centred at 480 cm^−1^, signature of the a-Si:H structure^21^. This Raman band at 480 cm^−1^ may be used for direct evaluation of the a-Si:H film thickness. However, due to the sharp and intense c-Si related Raman band at 520 cm^−1^, the amorphous signal from the thin a-Si:H overlayers is difficult to detect and the sensitivity of such a technique is insufficient to probe the few nm thick a-Si:H layer. Therefore we rather focused on the intensity of the c-Si related band.

Raman spectra were collected point-by-point for the flat c-Si wafer with a-Si:H stripes on a 40 × 40 mm^2^ large square grid, using a step size of 200 μm in both *x* and *y* directions. At every point the Raman signal is collected during 1 s using the 50× objective in order to acquire a sufficiently strong Raman signal. The Raman intensity map of c-Si is then calculated, integrating the c-Si Raman band spectral range from 505 to 535 cm^−1^ (see Fig. 1c). Since the 442 nm Raman excitation laser is strongly absorbed in the a-Si:H (absorption depth 1/α = 54 nm)^11^, even a layer of only several tens of nm thick attenuates its intensity significantly. Therefore the number of photons entering the underlying c-Si wafer is reduced and the Raman signal generated in c-Si wafer is proportionally lower. Moreover, due to the used back scattering detection geometry, the emitted c-Si Raman photons are partially absorbed in the a-Si:H film on their way back to the detector. Accordingly, the Raman signal detected from the c-Si wafer is reduced substantially by absorption in the overlaying thin a-Si:H film and the a-Si:H stripes are clearly visible on the c-Si Raman intensity map as long dark trenches, see map in Fig. 1c.

Similarly, the integrated c-Si Raman intensity map was measured for a rough c-Si wafer with a-Si:H stripes. On average, the approx. 2 μm high pyramids on the c-Si wafer make the use of the 50 × objective impossible, since the absolute c-Si Raman intensities detected under these conditions vary significantly with the local topography for bare c-Si wafer. This is a consequence of the micro-Raman system’s confocality, which is significantly reduced for objectives with lower numerical aperture, see discussion in Ref. 22. In order to obtain the same c-Si Raman signal intensities over the whole rough c-Si wafer, the 5× objective was used instead. Due to the low numerical aperture of this objective, the collection of the Raman signal is less efficient and the integration time was prolonged to 5 s at every point.

## Discussion

Since the detection path is the same for all the single Raman spectra measurements, the obtained contrast in c-Si Raman intensity (Fig. 1c) has to be given by the samples’ optical properties. Firstly, changes in laser light reflection may significantly affect the Raman signal for flat samples. In our specific case, the c-Si substrate refractive index (n) at the laser wavelength 442 nm is 4.7 (Ref. 23) and the one of a-Si:H layer varies with doping from 4.2 (for p-type films) to 4.8 (n-type)^11^. Therefore the reflection at the a-Si:H/c-Si interface given by the Fresnel equations is well below 0.5%. Consequently the interference effect may be neglected. Secondly, the surface reflection of p-type a-Si:H stripes is 3% lower compared to the c-Si part (see supporting information for measurement and detail discussion). Decreasing the laser reflection increases the c-Si Raman signal, which is opposite to the measured effect. Because of its insignificance (see detailed discussion in the supporting information), reflection effects will be neglected in the rest of the text. The main aim of this work is to measure the layer thickness on rough substrates, in this case no reflection changes were detectable by our set-up (well below 0.5% of relative reflection change).

This implies that the attenuation of the detected c-Si Raman signal is given by the absorption of the excitation laser and the back-scattered Raman photons in the a-Si:H film only. The wavelength of the Raman scattered light is 452 nm. This 10 nm wavelength shift does not change the optical properties significantly, therefore we will use average values of the absorption coefficients for the following calculations (the same approximation is used in the definition of the Raman collection depth^24^). The light intensity in light-absorbing media is described by the Lambert-Beer law:


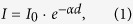


where *I_0_* is the initial intensity of the incident light, *α* is the absorption coefficient and *d* is the thickness of the absorbing film. In our case *I_0_* is the c-Si Raman signal intensity measured on an unprocessed, pristine wafer, which is directly proportional to the intensity of the excitation laser. Therefore, the c-Si Raman intensity measured at the a-Si:H stripe *I* is reduced by a factor of *e*^*−αd*^ due to absorption of the excitation laser in the a-Si:H film. Since the Raman signal is absorbed in the a-Si:H on the way back to the detector too, the total attenuation factor is *e*^*−2αd*^. From the formula (1) the thickness of the a-Si:H film is thus calculated as follows:


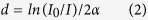


**Figure 2:**
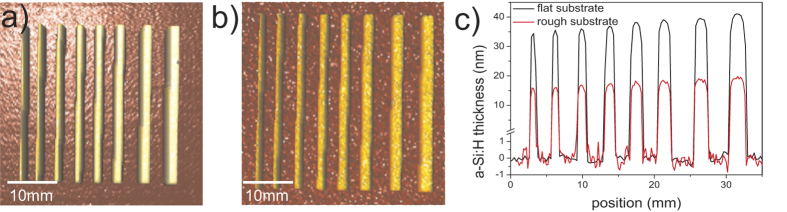
Maps of a-Si:H thickness based on Raman mapping measured on sample deposited on (**a**) flat c-Si wafer and (**b**) rough c-Si wafer (for height scale see the cross-section). Panel (**c**) gives cross-sections (line scans) of amorphous silicon stripes measured by Raman mapping on flat (black curve) and on rough (red curve) c-Si wafers. The scale below the y-axis break at 1.5 nm is zoomed-in to highlight the low noise-level of our measurements.

All measured Raman spectra were analyzed using the above-described method. The resulting thickness map of the a-Si:H stripes is plotted in Fig. 2 both for samples deposited on a flat and rough c-Si substrate.

Tens of nm thick a-Si:H stripes are clearly visible on both flat and rough c-Si substrates depicted in Fig. 2. The stripes are increasing in width from left to right, as the slit openings of the deposition mask were intentionally changed from 0.8 to 2.2 mm. For a detailed comparison of rough and flat samples, we extracted cross-sections profiles across all the a-Si:H stripes, see Fig. 2c).

Based on results from Fig. 2c we conclude that Raman spectroscopy can qualitatively probe well thickness profiles. In order to judge the absolute precision of Raman thickness determination, comparison with an independent measurement is essential. Therefore we have prepared a series of three p-doped a-Si:H films (without any pattern), deposited on flat c-Si substrates. Thicknesses of these films were measured by standard SE and by our Raman absorption technique, results are summarized in Table 1.

It is seen that thicknesses determined independently from SE and Raman are in very good agreement for all the test a-Si:H films. The SE measurement on these samples revealed consistently ~10% higher α values than those tabulated^11^, corresponding to lower p-doping levels. The Raman thicknesses were therefore calculated with the absorption coefficient obtained from SE measurements; knowledge of α is essential for precise determination of real thickness by Raman.

**Figure 3:**
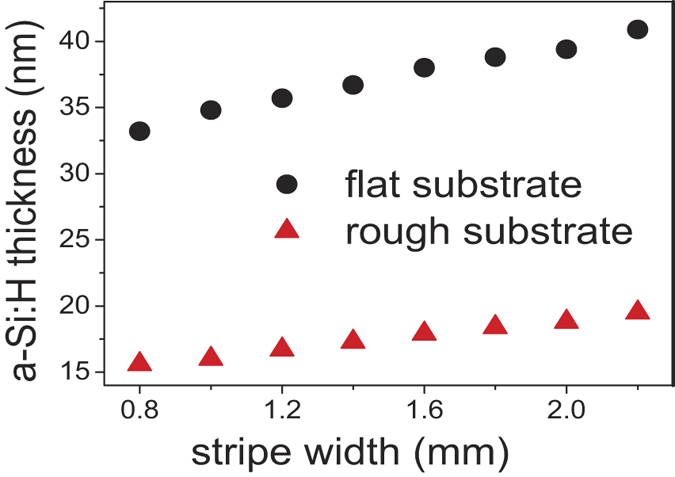
Thicknesses of the a-Si:H film measured in the middle of the stripes are plotted as a function of slit width on graph for both flat and rough c-Si substrates.

Our study reveals that the thickness of the amorphous stripes (measured at their centres) increases with the shadow mask slit width for both flat and rough silicon wafers (Fig. 3). In the probed range, the measured stripe thickness scales linearly with the shadow mask slit width. Using linear regression, the extrapolated thickness for a ‘zero’ slit width would be ~25 nm and 12 nm for flat and rough c-Si wafer respectively, where in reality it is of course 0 nm. This dependency indicates that the scaling trends become more complex towards smaller openings. However, in the probed range this simple linear dependency gives already a strong indication about the interaction of the plasma discharge with the mask. As the thickness of the a-Si:H film deposited without mask is even higher, we believe that the plasma discharge is pushed out of the slit area, leading to reduced deposition rates. Since the deposition conditions were identical for flat and rough substrates, it is reasonable to directly compare thicknesses of prepared a-Si:H stripes. The growth rates on flat substrates are higher in comparison with rough substrates. This is explained by the larger surface area in case of rough substrates; from the known shape of the pyramids on the rough silicon wafer (the top angle is 80°), we can calculate the rough-to-flat surface ratio to be about 1.75. Without shadow mask, co-deposition of planar films on flat and textured substrates is known to yield a thickness ratio of about 1.7^6^, which is very close to the calculated rough-to-flat surface ratio, indicative of the directionality of PECVD film growth. The measured deposition-rate ratio by Raman technique is 2.1±0.1 regardless of the slit widths. The higher measured value may be caused by inhomogeneous deposition on rough c-Si surfaces. The Raman technique averages the Raman intensity over the whole laser spot. Topologies with thinner a-Si:H parts contribute then proportionally more to the measured c-Si Raman intensity (due to the absorption exponential dependence on the film thickness). Therefore the calculated a-Si:H thickness is always underestimated in comparison with the real mean a-Si:H thickness.

In general, since Raman spectroscopy is an optical technique, it measures the *optical* thickness of thin films^11^, which may be significantly larger than their *physical* thickness in case of rough substrates, due to substantial light scattering. In the case of rough c-Si substrates, the incoming light is mainly trapped by geometrical effects. This is well proven by lower-than-expected a-Si:H thicknesses measured on rough c-Si, whereas light scattering should lead to higher values.

**Figure 4:**
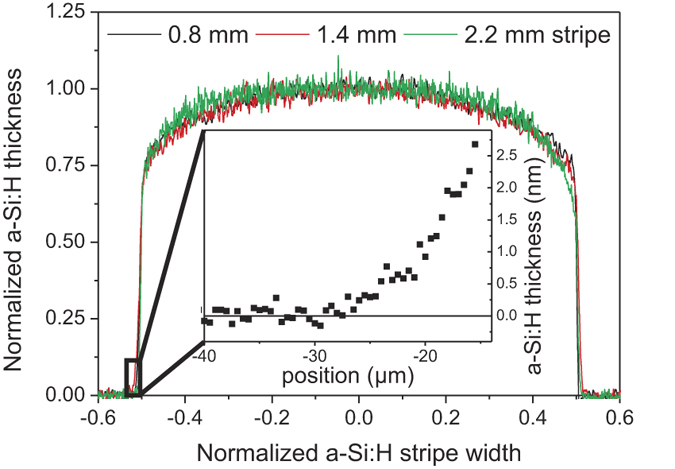
Line profiles of three a-Si:H stripes deposited on flat c-Si wafer (0.8, 1.6 and 2.2 mm width) normalized in height and width for an easier comparison of their shapes are plotted in the graph. The edge of the stripe was probed separately with a higher spatial resolution for the thickest stripe (2.2 mm), see the inset.

The detection limit of Raman-based thickness measurements is an important concern. A tentative estimation can be made based on the measured noise level in Fig. 2c). In principle, one may assume that the a-Si:H film is not deposited underneath the masked area, so its thickness there should be 0 nm. The measured noise level underneath this masked area (see Fig. 2c) is well below 1 nm, implying that an a-Si:H layer of this thickness should be detected. Unwanted deposition underneath the shadow mask, resulting in a spread of the a-Si:H stripe (so-called tailing), is suspected to deteriorate the solar cell performance^18^. To investigate to which extent this phenomenon actually occurs, all the stripes were measured with a high spatial resolution of 2 μm and the shape at the stripe edge was probed in even greater detail by a finer line scan with step of 500 nm for the sample deposited on flat c-Si. The calculated thickness profiles of three stripes (0.8, 1.6 and 2.2 mm width) are plotted in Fig. 4. For an easier comparison, these profiles are normalized in both height and width.

As can be seen from Fig. 4, the shape of the deposited a-Si:H stripe remains overall the same, regardless of the shadow mask slit width. If we define the edge of the stripe as the place with half of the maximal stripe thickness then the measured widths of the stripes perfectly match the nominal widths of the shadow masks (the largest discrepancy was 8 μm, well below realistic resolution of the measurement). More importantly, the thickness of the a-Si:H stripes is continuously decreasing towards the stripes edges. As a consequence, roughly 25% of the stripe is below 90% of the maximal stripe thickness. The hole and electron collecting a-Si:H selective contacts cannot be fully optimized in thickness, therefore its properties change over the a-Si:H stripe. We think that the plasma discharge during the PECVD process is pushed out of the stripe area, which leads to a lower SiH_x_ growth radical density at the edge region of the stripe and consequently to a local decrease in growth rate. Therefore, the electrical properties of one quarter of the stripe area are affected by the decreased film thickness, regardless of its width. Moreover the a-Si:H stripe thickness decreases exponentially with the distance from the slit edge. It is worth noting that the noise level of the estimated thickness is just few tenths of nm and a 0.2 nm thick a-Si:H layer is still detectable at a distance of 25 μm from the slit edge (see inset of Fig. 4). This type of inhomogeneities may lead to recombination losses in BC-SHJ devices and therefore has to be probed in detail.

We remark here that the profilometry method can be applied to a wide variety of material systems, well beyond the silicon heterojunction structures described here. In such a case, the refractive indices of used substrate and thin films may differ substantially. In order to correctly evaluate the layer absorbance, the substrate Raman signal as well as reflection of the laser beam may be measured simultaneously with a standard Raman set-up (as it was done for MoO_x_ layer on c-Si substrate, see supplementary information). Advanced optical modelling may be needed to interpret the changes in the absolute intensities of Raman and reflected signal, especially for flat samples with thicker films. Conversely, for films on substrates with a roughness comparable with those of our study, the reflection differs only very weakly for both deposited film and the substrate (see the supplementary information). Therefore reflection correction is in most cases not needed.

Another important application of this method is the determination of 2D materials thicknesses, i.e. number of monolayers for example in case of exfoliated graphene flakes. It is well known that every single graphene layer absorbs 2.3% of visible light^25^,^26^, independently of its wavelength. Usually the graphene 2D Raman band is used for determination of monolayer count, but its precision is very low already for three layers stack. Similar properties are found at the other 2D materials like for example MoS_2_^27^ or WS_2_^28^. Since the Raman spectroscopy is basic characterisation tool for all 2D materials, Raman profilometry is easy to implement.

The photoluminescence intensity may be used instead of the Raman signal, provided that its absolute intensity is homogenous over whole substrate. Since the photoluminescence signal is usually several hundred nm away from excitation wavelength, the difference in absorption coefficients has to be taken into account in formula 2. Multi-wavelength analysis may lead to even more precise results.

## Conclusions

We have developed a method based on Raman micro-spectroscopy for precise thickness profilometry (better than 0.5 nm), with very high lateral resolution up to 500 nm, applicable even on very rough substrates. Attenuation of the Raman intensity of a given substrate by absorption in an overlying thin film was used for its thickness detection. This technique was tested on few tens of nm thin a-Si:H stripes deposited on both flat and rough c-Si wafers, structures used in high-efficiency silicon heterojunction solar cells. Thickness mapping over several cm large areas with sub-nanometer resolution was introduced. Precision of Raman absorption thickness measurement was confirmed by independent thickness evaluation from spectroscopic ellipsometry. Observed thickness inhomogeneities, mainly the diminution at the stripe edges and tailing below the shadow mask was clearly documented; consequential restrictions for final heterojunction solar cells were discussed.

## Additional Information

**How to cite this article**: Ledinský, M. *et al*. Profilometry of thin films on rough substrates by Raman spectroscopy. *Sci. Rep.* **6**, 37859; doi: 10.1038/srep37859 (2016).

**Publisher's note:** Springer Nature remains neutral with regard to jurisdictional claims in published maps and institutional affiliations.

## Supplementary Material

Supplementary Information

## Figures and Tables

**Table 1:** Comparison of a-Si:H thicknesses measured by SE and Raman spectroscopy.

Sample	SE thickness (nm)	Raman (nm)
1	8.7 ± 1	10.4 ± 1
2	16 ± 1	16.1 ± 1
3	32.1 ± 2	30.9 ± 2
