# Supplementary material for: Profilometry of thin films on rough substrates by Raman spectroscopy
Source: Sci Rep. 2016 Dec 6;6:37859. doi: 10.1038/srep37859 (PMC5138622; doi:10.1038/srep37859)
Supplement: Supplementary Information [file srep37859-s1.pdf]

Profilometry of thin films on rough substrates by Raman spectroscopy

Martin Ledinský,[1,a)] Bertrand Paviet-Salomon,[2] Aliaksei Vetushka,[1] Jonas Geissbühler,[2] Andrea Tomasi,[3] Matthieu Despeisse,[2] Stefaan De Wolf,[4] Christophe Ballif[2, 3] and Antonín Fejfar[1]

[1] *Laboratory of Nanostructures and Nanomaterials, Institute of Physics, Academy of Sciences of the Czech Republic, v. v. i., Cukrovarnická 10, 162 00 Prague, Czech Republic*

[2] *PV-Center, Centre Suisse d'Électronique et de Microtechnique, Rue Jaquet-Droz 1, CH-2002 Neuchâtel, Switzerland*

[3] *École Polytechnique Fédérale de Lausanne (EPFL), Institute of microengineering (IMT), Photovoltaics and Thin Film Electronics Laboratory, Rue de la Maladière 71b, CH-2000 Neuchâtel, Switzerland*

[4] *King Abdullah University of Science and Technology (KAUST), KAUST Solar Center (KSC), Thuwal, 23955-6900, Saudi Arabia*

Supplementary information

Contrast in local reflection

In this part we discuss the role of reflection on the a-Si:H film and the flat c-Si substrate. Since the total reflection of a-Si:H depends on the refraction index n (and therefore on the effective doping level of a-Si:H) and on the thickness of the a-Si:H layer, its evaluation is a complex problem. That is why we have measured reflection directly by detecting reflected laser light intensity (signal at zero Raman shift, see fig. S1).

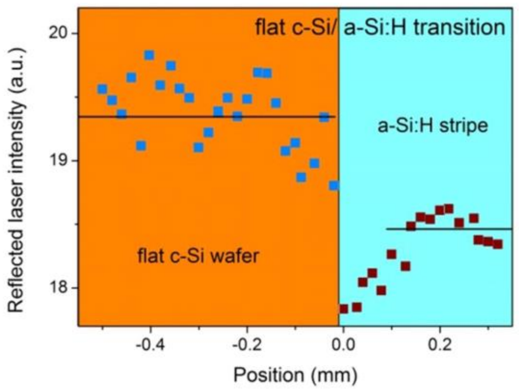

Figure S1: Intensity of reflected laser light measured across the transition of the flat c-Si wafer and the a-Si:H stripe. The first tens nm of the a-Si:H stripe have lower reflection most probably

due to higher roughness of thin silicon layer (may contain microcrystalline silicon fraction). This part was not taken into account. Lines are guides for the eyes only.

The c-Si reflection at 442 nm was calculated from known index of refraction (n = 4.7) to R_c = 0.42. The laser reflected light detected on the a-Si:H stripe was 4 % lower in comparison with the c-Si part. It means that the corresponding reflection of the a-Si:H stripe is R_a = 0.40. If we apply this correction, the a-Si:H stripe thickness will slightly decrease. For 40 nm thick layer (as measured on the widest a-Si:H stripe) the reflection correction will be less than 1 nm. Since the relative change is roughly 2 % only, we decided to keep the evaluation procedure straightforward and do not take in to account the minor reflection effect.

Moreover we have applied this method on MoO_x stripes deposited through the same mask by using thermal evaporation of MoO_3 powder [1] on flat and textured c-Si wafers. In contrast to the a-Si:H stripes, the index of refraction for the MoO_x layer and the c-Si substrate is very different (at 442 nm n = 2.3 [2]). Therefore the measured c-Si Raman intensity distinctly depends on the sample reflection. And as the absorption of this film is rather weak, its role will be minimal. This reasoning is well validated by results in figure S2.

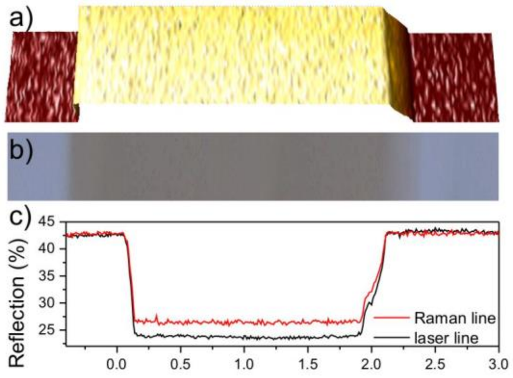

Figure S2: Map of the absolute c-Si Raman intensity over 2 mm wide MoO_x stripe a) (scale: min = 35 and max = 70). Optical image of probed part b) shows the antireflection effect of the MoO_x layer. Graph c) compares reflection directly measured at laser line and calculated from Raman intensity change (assuming non-absorbing film and no scattering at the surface).

Presence of the MoO_x layer leads to strong increase of the c-Si Raman signal, as we can see from the Raman map on fig. S2. From the white light optical image (Figure S2.b) we can conclude that the stripe reflection is lower, as the MoO_x layer serves as an antireflection coating. Thanks to the lower reflectivity more photons enter the c-Si substrate and therefore more Raman photons are created - Raman signal increases. The laser reflection ("Raman" signal at 0 cm^{-1}) was measured directly together with the c-Si Raman intensity. Assuming no absorption in MoO_x layer, local reflection was calculated from the Raman intensity data as well (Raman intensity proportional to squared transmission - to the laser intensity entering the c-Si wafer and the probability that the Raman photon escape the sample). Reflection is in both cases (for Raman line and laser line)

calibrated by the known value for a flat c-Si wafer. We find very good correlation between calculated and directly measured reflection values proving our concept. Less than 3% discrepancy may be ascribed to the scattering on the sample surface and not fully negligible absorption in the MoO_x film.

The same measurement was performed on the MoO_x stripes deposited on textured c-Si wafer. The main difference compared to the deposition on flat wafer is very small contrast in absolute Raman intensity on MoO_x stripe and plain c-Si wafer. The pyramids on the wafer surface serve for effective antireflection, therefore the additional contribution of MoO_x layer is roughly 6% relative only (see Fig. S3).

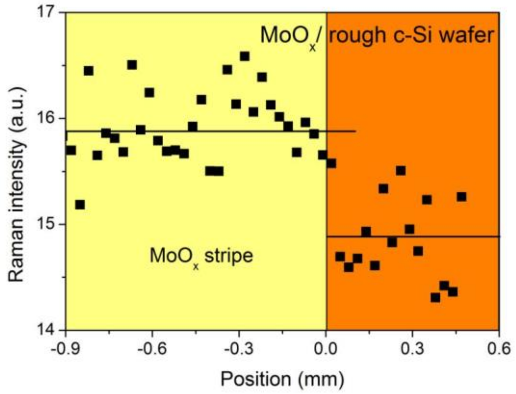

Figure S3: Intensity of the reflected laser light measured across the transition from flat c-Si wafer to the area of MoO_x stripe. Lines are guides for the eyes only.

Within this precision we can conclude that for the main application – the thickness measurement of layer deposited on rough substrate - there is no need to apply reflection corrections. For flat samples both the reflection and Raman signal should be detected in order to interpret the results correctly.

[1] Jonas Geissbühler, Jérémie Werner, Silvia Martin de Nicolas, Loris Barraud, Aïcha Hessler-Wyser, Matthieu Despeisse, Sylvain Nicolay, Andrea Tomasi, Bjoern Niesen, Stefaan De Wolf and Christophe Ballif 22.5% efficient silicon heterojunction solar cell with molybdenum oxide hole collector. Appl. Phys. Lett. **2015** 107, 081601

[2] L. Lajaunie, F. Boucher, R. Dessapt, and P. Moreau. Strong anisotropic influence of local-field effects on the dielectric response of α-MoO3, Phys. Rev. B **2013** 88, 115141
